# Arteriovenous fistulae after renal biopsy: diagnosis and outcomes using Doppler ultrasound assessment

**DOI:** 10.1186/s12882-017-0786-0

**Published:** 2017-12-20

**Authors:** R. Haridian Sosa-Barrios, Victor Burguera, Nuria Rodriguez-Mendiola, Cristina Galeano, Sandra Elias, Gloria Ruiz-Roso, Sara Jimenez-Alvaro, Fernando Liaño, Maite Rivera-Gorrin

**Affiliations:** 1 Nephrology Department. Ramon y Cajal University Hospital, Madrid, Spain; 2 Alcala University, Madrid, Spain; 3 Spanish Group of Diagnostic and Interventional Nephrology, Spanish Society of Nephrology, Madrid, Spain; 4 Red de investigacion renal (REDinREN), ISCIII (ERC 10 RD12/0021/0020) Nephrology, Madrid, Spain; 5 Instituto Ramon y Cajal de Investigacion Sanitaria (IRYCIS), Madrid, Spain

**Keywords:** Renal biopsy, Diagnostic and interventional nephrology, Renal ultrasound, Doppler scan, Arteriovenous fistula, Post biopsy complication

## Abstract

**Background:**

Percutaneous renal biopsy (PRB) is an important technique providing relevant information to guide diagnosis and treatment in renal disease. As an invasive procedure it has complications. Most studies up to date have analysed complications related to bleeding. We report the largest single-center experience on routine Doppler ultrasound (US) assessment post PRB, showing incidence and natural history of arteriovenous fistulae (AVF) post PRB.

**Methods:**

We retrospectively analysed 327 consecutive adult PRB performed at Ramon Cajal University Hospital between January 2011 and December 2014. All biopsies were done under real-time US guidance by a trained nephrologist. Routine Doppler mapping and kidney US was done within 24 h post biopsy regardless of symptoms. Comorbidities, full blood count, clotting, bleeding time and blood pressure were recorded at the time of biopsy. Post biopsy protocol included vitals and urine void checked visually for haematuria.

Logistic regression was used to investigate links between AVF, needle size, correcting for potential confounding variables.

**Results:**

46,5% were kidney transplants and 53,5% were native biopsies. Diagnostic material was obtained in 90,5% (142 grafts and 154 native). Forty-seven AVF’s (14.37%) were identified with routine kidney Doppler mapping, 95% asymptomatic (*n* = 45), 28 in grafts (18.4%) and 17 natives (9.7%) (*p*-value 0.7). Both groups were comparable in terms of comorbidities, passes, cylinders or biopsy yield (p-value NS). 80% were <1 cm in size and 46.6% closed spontaneously in less than 30 days (range 3–151). Larger AVF’s (1–2 cm) took a mean of 52 days to closure (range 13–151). Needle size was not statistically significant factor for AVF (p-value 0.71).

**Conclusions:**

Contrary to historical data published, AVF’s are a common complication post PRB that can be easily missed. Routine US Doppler mapping performed by trained staff is a cost-effective, non-invasive tool to diagnose and follow up AVF’s, helping to assess management.

## Background

Renal biopsy is one of the most important tools in Nephrology, defining diagnosis as well as guiding prognosis and treatment, both in native and transplanted kidneys [1]. There are different options to perform a kidney biopsy. Percutaneous real-time ultrasound guided technique (PRB) is one of the most used worlwide due to its safety profile [2–4]. Like all invasive procedures it has complications, mainly haematuria, perirrenal haematomas, active bleeding and urinary tract obstruction due to clots [5]. The complication rate should be around 5% or less, and can be minimised with a proper workup pre PRB and planning of technique [6].

Arteriovenous fistulae (AVF’s) are considered a rare complication post PRB in the literature up to now [7–10] reporting a variable incidence ranging from 3 to 5% in native (NK) to 10–16% in transplanted kidneys (TK) [8–10]. Most articles refer to symptomatic complications, mainly bleeding related [4, 5, 11], as there aren’t any routine post PRB imaging assessment protocols. In our centre, all nephrologists are capable to execute PRB, ultrasound and Doppler assessment of NK and TK [12, 13], with a mean of 100 biopsies, 2200 US and 430 Dopplers per year. Routine ultrasound imaging with Doppler evaluation of the biopsied kidney is performed within 24 h of the procedure, regardless of symptoms, and if any complication is detected a weekly US and Doppler follow up is done for the first month and monthly thereafter until resolution.

To our knowledge, this is the first report on ultrasound and Doppler mapping of the biopsied kidney, both native and transplanted, showing incidence and natural history of AVF’s in the era of real-time US guided PRB.

## Methods

We analysed 327 PRB done in our center in adults (>15 years old) from January 2011 until December 2014, both included. Data on age, gender, blood tests, blood pressure, comorbidities and renal function were collected prospectively. All PRB’s were performed by an experienced consultant nephrologist or a trainee under the direct supervision of a consultant nephrologist. Real-time US guidance was done by the nephrologist in all cases, with a previous simulation to select punction area and assess patient tolerance to procedure.

This study was approved by the Institutional Review Board at University Hospital Ramon y Cajal. As collected data were derived from routine clinical practise no further consents were obtained.

Statistical analysis was performed using SPSS 20 package (©SPSS Inc., Chicago, IL) with significance performed with t-test or Mann-Whitney test, and Fisher’s exact test for categorical data. Logistic regression analysis was done to evaluate risk factors and confounding variables. Data are reported as mean ± standard deviation (SD) or range and *p*-value <0.05 was considered significant.

### Previous work up

Full blood count (FBC), biochemistry and clotting (fibrinogen, prothrombin time, partial thromboplastin time) including bleeding time (colagen-epinephrin and colagen-ADP) were checked before PRB. In our practice, haemoglobin >9 g/L, normal clotting and bleeding time, and blood pressure < 160/90 are required to proceed. Single kidneys and patients unable or unwilling to co-operate were dismissed. If patients were on oral anticoagulation or platelet aggregation inhibitors those were suspended or switched to low molecular weight heparin (LMWH) a week before procedure, not being administered 24 h beforehand.

### Procedure

Real-time US guided PRB was done in all cases by the nephrologist with full aseptique technique using local anesthesia (lidocaine 1% for TK and 2% for NK) along the needle insertion tract under real-time US guidance. No sedatives were given. Biopsies of NK were done in prone position and TK in supine, targeting left lower renal third for NK and cranial third for TK.

A 14 Gauge (14G) for NK or 16 Gauge (16G) for TK automated spring fired needle was used (©Acecut TSK, Japan) with a biopsy probe adaptor (64° needle-skin angle for NT and 54° for TK), aiming to obtain 2 tissue cores.

All PRB required admission to hospital early on the day of procedure, remaining nil by mouth for at least 6 h prior to it. Twenty four hour bed rest post biopsy was mandatory and close monitoring of signs and symptoms of complication was done. Blood pressure, heart rate and urine void to look for macroscopic haematuria were checked every 15 min for the first hour, hourly for 3 h and every 6 h thereafter.

**Fig. 1:**
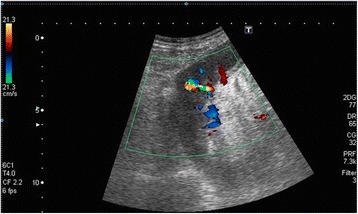
Color Doppler ultrasound image of an AV fistula in a transplant kidney. The image is from a convexe transducer showing the upper pole. The fistula is evident as a coloured area < 1 cm from the renal capsule. Red, yellow, green and blue are seen within it, suggesting high velocity, disturbed flow

**Fig. 2:**
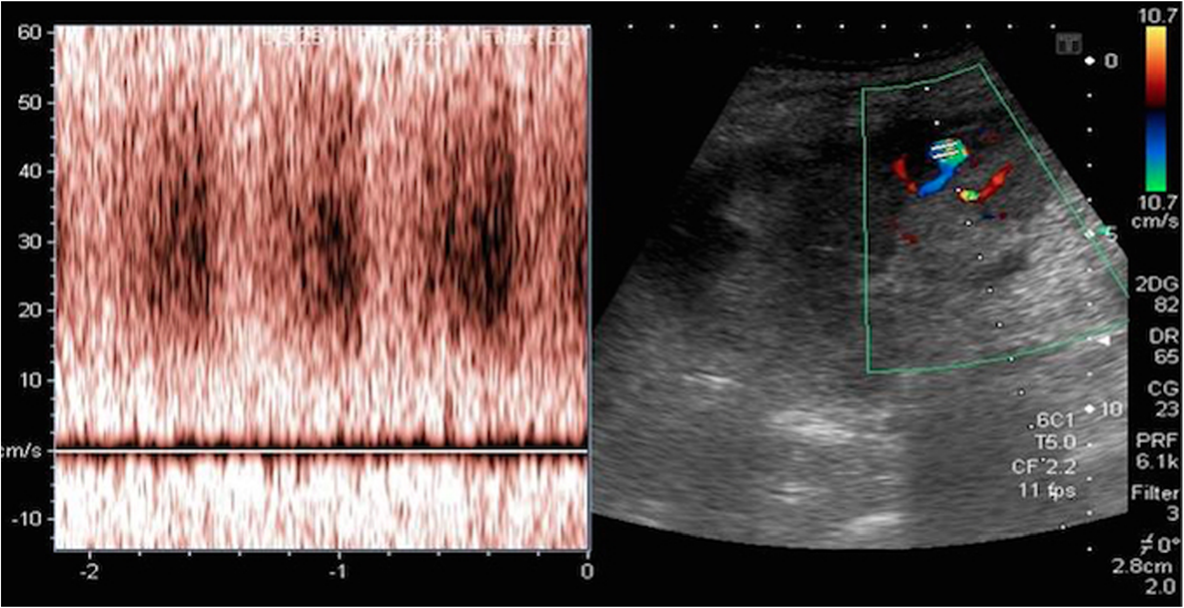
The flow waveform is characterized by high velocities throughout with reduced systolic-diastolic difference and high, arterialized venous flow on spectral analysis

The day after procedure, a routine renal US and Doppler mapping of the biopsied kidney was done by a consultant nephrologist or a trainee under direct supervision of a consultant nephrologist, using a ® Toshiba Xario 600 ultrasound device with a 3.5 MHz curvilinear probe. AVF’s were diagnosed with the following criteria:

- Colour aliasing (confetti-like pattern) in a circumscribed area of the renal parenchyma showing turbulent flow (Fig. 1).
- High velocity arterial flow with reduced systolic-diastolic difference and high, arterialized venous flow on spectral analysis (Fig. 2).

FBC was repeated the day after biopsy if no issues arose. Further intervention was decided as per clinician assessment of the situation at the time.

## Results

The clinical and laboratory characteristics of the patients in the two groups are compared in Table 1. Three hundred twenty seven biopsy procedures were analysed, 152 TK (46.5%) and 175 NK (53.5%). 64% were male and 36% women, mean age 55 ± 15 years. 73% were hypertensive and 18% had diabetes mellitus (either type 1 or 2). Statistical analysis showed no difference on baseline characteristics between the transplant and native kidneys groups, except for male gender and hypertension in the transplant group (*p*-value 0.024).

**Table 1:** Baseline characteristics of NK and TK patients undergoing PRB with and without AVF

	NK	NK AVF	TK	TK AVF
	no AVF		no AVF	
No. pts	158	17	124	28
Age	57.8 ± 17.4	57.68 ± 19.81	51.8 ± 11.6	51.04 ± 8.33
Gender (%male)	57.6	34.4	71.7	65.5
Diabetes (%)	17.6	11.7	19.5	10.7
Systolic BP (mmHg)	139.6 ± 11	139.3 ± 9	152.5 ± 12	150.2 ± 8
Diastolic BP (mmHg)	79.8 ± 8	79.3 ± 9	84.6 ± 6	86.1 ± 4
Hypertension (%)	60.8	64.7	85.8	85.7
Passes	1.32	1.39	1.45	1.33
Cores	1.11	1.14	1.17	1.11
Glomeruli	9.36	8.46	10.2	11.4

Average passes were 1.3 (range 1–4, no blank passes) with 1.33 average cilinders (range 1–3). Adequate diagnostic material was obtained in 90.52% (as per BANFF criteria for TK [14]), with 9.77 average glomeruli (range 0–45). We found no difference in biopsy yield between the two different needle sizes (*p*-value 0.6).

Forty seven AVF’s were identified, showing an incidence of 14.37%, 95% were asymptomatic. Two of them required supraselective catheter embolization due to symptoms in the first 12 h post biopsy (haemodynamic instability and active bleeding, both with good outcome preserving kidney function) and were excluded from further analysis after checking both AVF’s were closed.

Of the remaining 45 asymptomatic AVF’s detected in the first 24 h post biopsy, 28 were TK and 17 in NK (18.4% vs 9.7%, p-value 0.07). Gender related, 31% were women and 69% men. Most AVF’s were <1 cm in size (80%, 24 TK and 12 NK), with only 1 AVF >2 cm (NK). Average time to closure was 25.57 days in TK (range 3–101) and 35.2 days in NK (range 3–151) without statistical significance. In less than 30 days 46.6% of AVF’s were closed, 95.4% by month 3 post biopsy.

None of them developed any symptoms related to the fistula (no macroscopic haematuria, cardiac failure or renal dysfunction), nor did they grow in size during follow up.

Multivariate analysis using logistic regression was performed to determine which features at baseline and technique variables were predictive of AVF following renal biopsy, including those more frequently studied in the literature (hematocrit pre PRB, serum creatinine, race). In our study, none of those neither needle size, blood pressure or kidney type were statistically significant factors for developing an AVF post biopsy (*p*-values 0.7, 0.1 and 0.7 respectively).

## Discussion

Renal biopsy has become a key tool in Nephrology, providing diagnosis, prognosis and, therefore, guiding treatment options [1]. With time, the renal biopsy procedure has evolved from blind approach using a Tru-Cut to ultrasound guided using spring loaded automated biopsy needles [2, 3], known as ultrasound guided percutaneous biopsy technique (PRB). PRB’s can be done with prior ultrasound (US) scanning to locate kidneys or real-time US guidance during the whole procedure.

It used to be a skill included in the Nephrology portfolio, but nowadays in many centers other specialties are in charge of PRB’s. At present, more nephrologists aim to perform PRB’s to reduce waiting times, optimise resources and provide complete care for patients [15], helping decision-making regarding the most cost-effective course of action. Appropriate training in PRB technique should be warranted to nephrologists, implementing programs to do so aiming to minimize complication rate with adequate biopsy yield [4–17].

As an invasive procedure PRB’s have complications, but its safety profile has improved dramatically after the introduction of US location and guidance [3–5]. Most studies focus on bleeding related ones: intraparenchymatous and perinephric haematomas, active bleeding requiring intervention and obstruction due to clots [2, 4, 5, 11].

Arteriovenous fistulae (AVF) are considered rare and its clinical impact varies in the few reports up to date [4, 5, 7–10, 19], referring mainly to AVF’s post biopsy in transplanted kidneys (Table 2). Up to now, none of them using real-time US guided PRB technique done by nephrologists in both native and transplanted kidneys.

**Table 2:** Post renal biopsy complication studies characteristics

Study	Journal	Year	N. Pts	Kidney Type	Technique	Avf Considered	Routine US Post PRB	Incidence	Outcome	Done By
Deane C et al	Urol Radiol	1992	126	Transplant	Not specified	Yes	Yes	17.5%	63% closure	Not specified
Merkus JW et al	Br J Surg	1993	62	Transplant	Not specified	Yes	Within 2w post PRB	10%	Closure/asymp.	Not specified
Brandenburg VM et al	Clin Nephrol	2002	72	Transplant	US guided	Yes	4-6 h post PRB	17%	50% Closure	Nephrologist
Whittier WL et al	J Am Soc Nephrol	2004	750	Native	US guided by Radiologists	Yes	No	0.4%	Not specified	Nephrologist
Shidham GB et al	Nephrology	2005	645	Native	US to locate	Yes	Inmediate post PRB	0.64%	Embolized	Not specified
Maya ID et al	Semin Dial	2007	129	Native	Blind 64/US 65	No	Inmediate post PRB	–	–	Rad/Nep
Tondel C et al^a^	Clin J Am Soc Nephrol	2012	9288	Native	US guided by Radiologists	No	No	–	–	Rad/Nep
Korbet SM et al	Am J Nephrology	2014	1055	Native	US guided by Radiologists	No	No	–	–	Rad/Nep
Lubomirova M et al^a^	OA Maced J Med Sci	2015	516	Both	US guided/Blind	Yes	1 day post PRB	0.8%	–	Nephrologist

^a^Multicenter. Rad = Radiology. Nep = Nephrology

The majority of AVF’s were diagnosed due to presenting symptoms, either bleeding related as an incidental finding or due to haemodynamic changes or loss of renal function. No routine US and Doppler assessment is performed in most centers post biopsy [18], or they scan for bleeding related ones [19, 20].

Our center has a wide experience in Diagnostic and Interventional Nephrology [12, 13]. All medical staff are trained on ultrasound scanning and Doppler mapping of both native (NK) and transplanted kidneys (TK). We have performed kidney ultrasound and Doppler since 1995, with a mean of 2200 US, 430 Doppler scans and 100–120 biopsies per year.

We have developed a simulation tool and live animal model to teach medical Nephrology staff the PRB technique [16] before practicing on patients. Kidneys are scanned with transverse and longitudinal images to evaluate cortical thickness, echogenicity and rule out structural abnormalities prior to biopsy. The day post procedure an US scan and Doppler mapping is done in all cases regardless of symptoms, by the same experienced team.

If a complication post PRB is detected, a follow up plan is established. In case of AVF’s we perform weekly US and Doppler for the first month, and monthly thereafter until closure.

We report the natural history of AVF’s in transplanted and native kidneys, showing higher incidence than reported in previous studies [5, 7–10]. A vast majority do not require any intervention and 95.4% of them close spontaneously within 3 months post biopsy. Most cases are asymptomatic and no usual related symptoms were seen, like gross haematuria, haemodynamic changes due to high shunt flow or loss of kidney function.

US and Doppler evaluation of NT and TK before and after PRB has been implemented in our department, helping to minimize technique risks. It is an inexpensive, reproducible and highly effective diagnostic tool, helping to guide post biopsy AVF’s management. In our experience, it is relevant to diagnose AVF’s even when a patient is asymptomatic, as it may develop symptoms with time and new clinical findings could be attributed to them, needing treatment [21, 22].

We failed to demonstrate needle size, kidney type or hypertension are risks factors to develop an AVF post biopsy, like reported in other studies related to bleeding complications post biopsy [2–5, 7–11].

To our knowledge, this is the first report on incidence and natural history of post biopsy AVF’s, both in native and transplanted kidneys, since the introduction of real-time ultrasound guidance performed by nephrologists.

## Conclusions

Post biopsy AVF’s are frequent and mainly asymptomatic, but can evolve to symptomatic pathology. Routine use of US and Doppler scanning following renal biopsy, whether native or transplanted kidneys, is essential to identify and standardize AVF’s management.

## Abbreviations

AVF: Arteriovenous fistulae
FBC: Full blood count
LMWH: Low molecular weight heparin
NK: Native kidney
PRB: Percutaneous renal biopsy
SD: Standard deviation
TK: Transplanted kidney
US: Ultrasound
